# Consistency Analysis in Medical Empathy Intervention Research

**DOI:** 10.3390/ijerph191710904

**Published:** 2022-09-01

**Authors:** Meng-Lin Lee, Ton-Lin Hsieh, Chih-Wei Yang, Jou-Chieh Chen, Yu-Jeng Ju, I-Ping Hsueh

**Affiliations:** 1 Division of Cardiovascular Surgery, Department of Surgery, Cathay General Hospital, Taipei 10630, Taiwan; 2 School of Occupational Therapy, College of Medicine, National Taiwan University, Taipei 10055, Taiwan; 3 Department of Medical Education, National Taiwan University Hospital, Taipei 10002, Taiwan; 4 Department of Emergency Medicine, National Taiwan University Hospital, Taipei 10002, Taiwan; 5 Department and Graduate Institute of Medical Education and Bioethics, College of Medicine, National Taiwan University, Taipei 100233, Taiwan; 6 Department of Physical Medicine and Rehabilitation, National Taiwan University Hospital, Taipei 10002, Taiwan

**Keywords:** definition, empathy, intervention, medicine, randomized controlled trial, clinical competence, medical education

## Abstract

Various studies have examined the effectiveness of interventions to increase empathy in medical professionals. However, inconsistencies may exist in the definitions, interventions, and assessments of empathy. Inconsistencies jeopardize the internal validity and generalization of the research findings. The main purpose of this study was to examine the internal consistency among the definitions, interventions, and assessments of empathy in medical empathy intervention studies. We also examined the interventions and assessments in terms of the knowledge–attitude–behavior aspects. We conducted a literature search for medical empathy intervention studies with a design of randomized controlled trials and categorized each study according to the dimensions of empathy and knowledge–attitude–behavior aspects. The consistencies among the definitions, interventions, and assessments were calculated. A total of 13 studies were included in this study. No studies were fully consistent in their definitions, interventions, and assessments of empathy. Only four studies were partially consistent. In terms of knowledge–attitude–behavior aspects, four studies were fully consistent, two studies were partially consistent, and seven studies were inconsistent. Most medical empathy intervention studies are inconsistent in their definitions, interventions, and assessments of empathy, as well as the knowledge–attitude–behavior aspects between interventions and assessments. These inconsistencies may have affected the internal validity and generalization of the research results.

## 1. Introduction

Empathy has been recognized as an important element in the doctor–patient relationship and medical practice [1]. Physicians are considered to provide good quality of care when they are “empathetic” toward patients [2,3]. Various definitions of empathy exist in the literature [4,5,6,7,8]. Although there is no consensus regarding the definition, it has been proposed that empathy consists of at least one of three dimensions: cognitive (thinking), affective (feeling), and behavioral (acting) [1]. Cognitive empathy can be defined as understanding others’ thoughts or thought processes. Affective empathy can be defined as understanding others’ feelings or emotional states. Behavioral empathy can be defined as performing certain actions or behaviors that show understanding of others.

Many empathy intervention studies have attempted to increase empathy in medical education [9,10,11]. Among them, various measures have been used to assess the effectiveness of different interventions [9,10,11]. Due to the various definitions of empathy, it has been recognized that certain inconsistencies exist between the definition and the concepts that are assessed by the measures of empathy [1,12]. For instance, the Jefferson Scale of Empathy (JSE), a common measure to assess empathy, consists mostly of “cognitive” items with no items on action [1]. It was developed by Hojat and colleagues, who defined empathy as “a predominantly cognitive (as opposed to affective or emotional) attribute that involves an understanding (as opposed to feeling) of patients’ experiences, concerns, and perspectives combined with a capacity to communicate this understanding” [13]. When the JSE was used to evaluate empathy concepts in Hojat et al.’s studies, the operationalization was not matched to its definition due to the lack of the behavioral dimension (a capacity to communicate this understanding). A systematic review even showed that only 14% of medical educational research defined and operationalized empathy consistently [1]. However, the consistency among the definitions, interventions, and assessments in medical empathy intervention studies remains largely unknown.

In addition, the knowledge–attitude–behavior (KAB) model has been advocated to be beneficial for health-related educational interventions. The KAB model is based on the theory that knowledge should be developed first, followed by changes in attitude and subsequently in behavior [14,15,16]. To the best of our knowledge, no studies thus far have examined the consistency in the medical empathy intervention studies by these approaches.

Therefore, there were two purposes of this study. The first purpose was to examine, in the current medical empathy intervention studies with a randomized controlled trial (RCT) design, the consistency among the sections describing the definitions, interventions, and assessments in terms of the cognitive, affective, and behavioral dimensions of empathy. The second purpose was to determine, in the same literature, the agreement between the sections describing the interventions and assessments in terms of the aspects of KAB. The findings should inform empathy intervention studies in the future.

## 2. Methods

Our research method was generally based on the manual for evidence synthesis from the Joanna Briggs Institute [17].

### 2.1. Search Strategy and Study Selection

To review journal articles on empathy interventions on medical doctors or students, we searched for relevant publications in the PubMed and Scopus databases (see Appendix A for details). Publications that were published from 1 January 2000 to 31 December 2021 were retrieved. In addition, we also did manual searches in the reference lists in the review articles that were retrieved from the database searches.

The retrieved studies from the database and manual searches were screened with the following eligibility criteria: (1) study samples that were based on medical doctors/students, (2) studies that were aimed at enhancing empathy, (3) studies with a randomized control trial (RCT) design, (4) outcome variables of intervention including empathy or empathic performance, and (5) original research studies.

**Figure 1:**
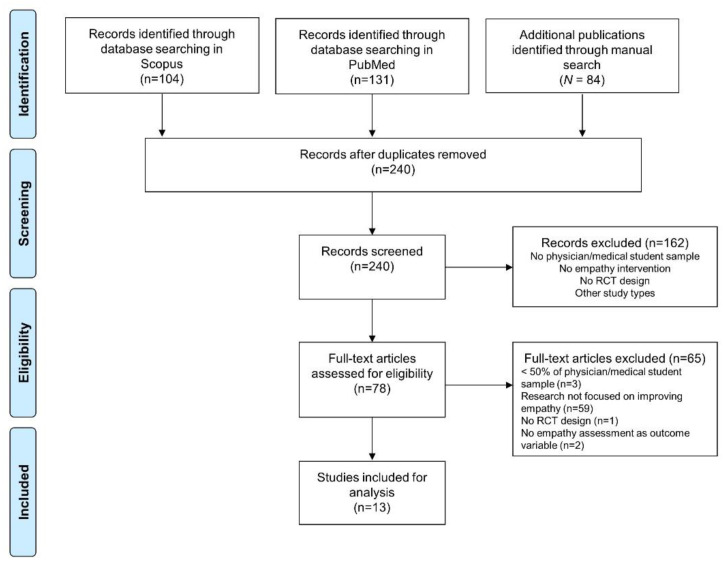
Flow chart of the search process.

The titles and abstracts of the retrieved studies were screened according to the eligibility criteria. Then, the full texts of the selected studies were examined to confirm their eligibility. The searching and screening procedures are presented in Figure 1.

### 2.2. Data Extraction

#### 2.2.1. Dimensions of Empathy in Definition, Intervention, and Assessment Sections

We classified all of the included studies by empathy dimensions (i.e., cognitive, affective, and behavioral) in three different sections (i.e., definition, intervention, and assessment). Ambiguity and uncertainty regarding categorizations of empathy dimensions were reviewed and discussed among the authors until agreement was reached.

For the conceptual definition of empathy, we extracted definitions that were either written explicitly (mostly in introduction sections) by the author(s) or quoted from other publications. For the interventions of empathy, we classified the intervention programs by their content that were explicitly described in the methods section, appendices or supplementary data. When multiple interventions were used in one study, all of the relevant interventions were considered for classification. For the assessment of empathy, we classified the assessments by the measures that were utilized in the studies. A measure was categorized into a certain dimension if at least three items or 1/3 of all of the items matched that dimension. For measures with only a single item, that single item’s dimension could represent the measure’s dimension. The categorization criteria of empathy dimensions in each section are detailed in Appendix B.

#### 2.2.2. Aspects of the KAB Model in Intervention and Assessment Sections of Empathy

We also examined whether the interventions and assessments of all of the included studies fulfilled the KAB model; that is, teaching and assessing empathy in terms of the knowledge, attitude, and behavior aspects.

For the intervention, we classified interventions by the contents that were explicitly mentioned in the articles (i.e., in methods, appendices, or supplementary materials). For the assessment, we considered a measure to comprise certain aspects of the KAB model if at least three items or 1/3 of all items were present. The categorization criteria of KAB aspects in both sections are detailed in Appendix C.

### 2.3. Data Analysis

We examined consistency in terms of the empathy dimensions among the definition, intervention, and assessment sections of the studies. In addition, the knowledge, attitude, and behavior aspects of the KAB model were also compared between the intervention and assessment sections.

#### 2.3.1. Consistency in Terms of Empathy Dimensions among Definition, Intervention, and Assessment Sections

The overall consistency and pairwise comparisons of the consistency between two specific sections of the studies (definition vs. intervention; intervention vs. assessment; definition vs. assessment) were calculated. Overall, consistency was defined as the numbers of dimensions that were constantly present in all three sections of the studies divided by the total numbers of dimensions that were available for comparison. When one dimension was absent in all three sections, that dimension was considered not applicable for comparison. Pairwise comparison of the consistency was defined as the numbers of dimensions that were constantly present in the two specific sections divided by the total number of dimensions that were available for comparison. When one dimension was absent in both sections, that dimension was considered inapplicable for comparison.

For example, if one study defined empathy as fulfilling cognitive, affective, and behavioral dimensions, with only the affective dimension fulfilled in the intervention section and only the behavioral dimension fulfilled in the assessment section, the percentage of consistency between the definition and intervention sections was calculated as 33% (1/3), the percentage of consistency between the intervention and assessment sections was 0% (0/2), and the percentage of consistency between the definition and assessment section was 33% (1/3). The overall consistency was 0/3 = 0%.

#### 2.3.2. Consistency in Terms of Aspects of the KAB Model between Intervention and Assessment Sections

Likewise, consistency in terms of the KAB aspects was calculated by the numbers of aspects that were constantly present across the intervention and assessment sections divided by the total number of aspects that were available for comparison. When one aspect was absent in both sections, that aspect was considered inapplicable for comparison. Therefore, the possible results of the percentage of consistency included 100%, 67%, 50%, 33%, 0%, and inapplicable (0/0).

## 3. Results

A total of 13 studies were included for analysis. The flow chart of the search process is presented in Figure 1. Details of the categorization of the empathy dimensions in the definition, intervention, and assessment sections are presented in Table 1.

### 3.1. Empathy Dimensions

#### 3.1.1. Definition

In the definition sections, seven studies mentioned cognitive dimensions of empathy, nine mentioned affective dimensions of empathy, and six mentioned behavioral dimensions of empathy. On the other hand, four studies did not define empathy in any dimension [27,34,40,48], while one study defined empathy in one dimension, three studies in two dimensions, and only five studies in all three dimensions.

#### 3.1.2. Intervention

In the intervention sections, two studies had interventions involving cognitive dimensions of empathy, two had interventions involving affective dimensions of empathy, and seven had interventions involving behavioral dimensions of empathy. Meanwhile, five studies had no interventions involving any dimension of empathy. The interventions included listening to an auditory hallucination [21], art-making [34], narrative medicine [44], Balint group training [38,44], and meditation [45].

#### 3.1.3. Assessment

In the assessment sections, five studies used measures involving cognitive dimensions of empathy, five used measures involving affective dimensions of empathy, and nine used measures involving behavioral dimensions of empathy. However, three studies did not use measures involving any dimension of empathy [21,34,44].

### 3.2. Consistency in Terms of Empathy Dimensions among Definition, Intervention, and Assessment Sections

The overall consistency and pairwise comparison of the consistency in terms of empathy dimensions are presented in Table 2.

#### 3.2.1. Overall Consistency

Among these 13 studies, none reached 100% overall consistency based on our categorization. There were four studies that were considered partially consistent, with the highest percentage being 67% in Riess et al.’s study [32], which involved affective and behavioral dimensions. The other three studies were 33% consistent [18,37,42], which involved the behavioral dimension only. The remaining studies had either no consistency at all, found in eight studies [21,23,27,38,40,44,45,48], or inapplicability for comparison, found in one study [34].

#### 3.2.2. Pairwise Comparison of the Consistency

In the comparisons of definition vs. intervention, no studies reached 100% consistency. There were five studies that had partial consistency, with two studies [32,42] having 67%, one study [23] having 50%, and two studies [18,37] having 33% consistency. The remaining studies had either no consistency at all, found in seven studies [21,27,38,40,44,45,48], or inapplicability for comparison, found in one study [34].

In the comparisons of intervention vs. assessment, three studies reached 100% consistency [18,37,48]. Interestingly, all three interventions were communication-associated workshops or educational courses, and all three corresponding measures involved the Empathy Communication Coding System (ECCS) [20]. Both interventions and measures fulfilled the category of the behavioral dimension. There were three studies that had partial consistency, with one study [32] having 67%, another one [42] having 50%, and the other [27] having 33% consistency. The remaining studies had either no consistency at all, found in four studies [23,38,40,45], or inapplicability for comparison, found in three studies [21,34,44].

In the comparisons of definition vs. assessment, only one study reached 100% consistency [32]. There were five studies that had partial consistency, with two studies [38,45] having 67% and three studies [18,37,42] having 33% consistency. The remaining studies had either no consistency at all, found in six studies [21,23,27,40,44,48], or inapplicability for comparison, found in one study [34].

### 3.3. Aspects of the KAB Model

The categorization of aspects of the KAB model in the intervention and assessment sections is presented in Table 1.

#### 3.3.1. Intervention

In the intervention sections, three studies [32,40,42] had interventions involving knowledge aspects. Surprisingly, none had interventions involving the attitude aspect, and seven studies [18,27,32,37,40,42,48] had interventions involving behavior aspects. Meanwhile, six studies had no interventions involving any aspects of the KAB model [21,23,34,38,44,45].

#### 3.3.2. Assessment

In the assessment sections, only one study [32] used a measure involving the knowledge aspect, eight studies [21,32,34,38,40,42,44,45] used measures involving attitude aspects, and nine studies [18,23,27,32,37,38,42,45,48] used measures involving behavior aspects. All studies had assessments involving at least one aspect of the KAB model.

### 3.4. Consistency in Terms of KAB Aspects between Intervention and Assessment Sections

The consistency of KAB aspects is detailed in Table 2. Among these 13 studies, four studies [18,27,37,48] reached 100% consistency based on our categorization of KAB aspects. Interestingly, all of them had full consistency involving only the behavior aspect. There were two studies that were considered partially consistent; one was Riess et al.’s study [32] (67%), and the other was Wündrich et al.’s study [42] (33%). The remaining seven studies had no consistency at all [21,23,34,38,40,44,45].

## 4. Discussions

To the best of our knowledge, this is the first attempt to examine medical empathy intervention studies with this unique and systematic approach. Results showed that internal contradictions in the definitions, interventions, and assessments of empathy were common in contemporary medical empathy intervention research. Hence, the effects of the associated empathy interventions demonstrated by the studies may have been underestimated due to the presence of inconsistency.

### 4.1. Empathy Dimensions

We found that the overall consistency in terms of empathy dimensions was poor in most studies. No studies reached 100% overall consistency, and only four had partial consistency [18,32,37,42]. The main contributing issue was the incompleteness in providing definitions, interventions, and assessments that corresponded with dimensions of empathy. There were four studies that did not provide a definition of empathy [27,34,40,48]. There were five study interventions that did not include any dimensions of empathy [21,34,38,44,45]. Furthermore, three studies did not use measures that assessed any dimensions of empathy [21,34,44]. As such, there was poor overall consistency.

During the analysis of overall consistency, the behavioral dimension was found to play an important role. In all four partially-consistent studies, the behavior dimension was present in every section and contributed to the overall consistency. The significance was further consolidated with the finding of 100% consistency based on the alignment of the behavioral dimension in the pairwise comparisons between the intervention and assessment sections in three studies [18,37,48]. Further examination of the contents of the three specific studies showed that all three interventions were communication-associated workshops or educational courses, and the corresponding measures were all involved with ECCS. Communication-associated educational activities and the corresponding measures for empathy-expressing behaviors were noted to be good matches based on our categorization of the behavior dimension in both sections. The important role of the behavioral dimension may be explained by the relatively accessible characteristics of empathy-expressing behaviors to be observed or quantified during assessment, in contrast to the rather abstract characteristics of the cognitive or affective dimensions of empathy.

### 4.2. Aspects of the KAB Model

General inconsistency was also evident from the comparisons of the intervention and assessment sections in terms of KAB aspects. About half of the studies (7/13) did not have any consistency at all. Further examination of the contents of these seven studies showed that the inconsistencies observed in six of them were due to mismatch of attitude aspects between the two sections (Table 2). The attitude aspect was not present in the interventions of these six studies, but all were assessed with the JSE. In other words, empathy measures that assess attitude are being used in intervention studies despite attitudes of empathy not being taught. This issue deserves further attention.

There were seven studies that intervened in the behavior aspect, while nine studies assessed it. The alignment of the behavior aspect was seemingly achieved in our analysis. Moreover, all four studies that reached 100% consistency in terms of the KAB aspects comprised only a single behavior aspect in both intervention and assessment sections. Therefore, the behavior aspect, reflecting the role of the behavioral dimension in the analysis in terms of the empathy dimension, has been an effective component contributing to the consistency among empathy studies.

### 4.3. Evaluation of Patient Satisfaction or Trust as an Outcome Measure

Among our included studies, patient satisfaction or trust was a commonly encountered outcome measure [23,27,48]. Based on our criteria, neither of these factors was considered as comprising any dimensions of empathy or KAB aspects. However, they may still play an important role in evaluating the effectiveness of an intervention. They are simply not variables for empathy measurement, although they may affect the physician–patient relationship in a broader context.

### 4.4. Recommendations

#### 4.4.1. An Ideal Design for Empathy Intervention Studies

An ideal study design should incorporate every dimension of empathy in every section and also every aspect of KAB, if included, in the corresponding sections.

In other words, the cognitive, affective, and behavioral dimensions are all indispensable in the definition, intervention, and assessment sections; meanwhile, the knowledge, attitude, and behavior aspects must also be included in the intervention and assessment sections. A study of such design would fulfill all the criteria of a fully consistent study and hence be believed to enhance the empathy of medical professionals to the best extent.

#### 4.4.2. Matching in Corresponding Sections

Other than the above-mentioned ideal design, based on our definition of consistency, a study should at least comprise of certain dimensions and aspects in every section of the study congruently. It would be prudent to approach the definition section first. Clarifying the definition of empathy as the initial step in the design of an empathy intervention study will help researchers develop the subsequent intervention methods and assessment measures correspondingly based on the pre-defined empathy dimensions. In addition, it would also be beneficial to align the intervention and assessment section in terms of KAB aspects to achieve an effective outcome. A study of such design would be internally consistent and valid.

#### 4.4.3. Real-World Concerns Regarding Already-Developed Measures

An empathy researcher needs to be cautious in applying the currently available measures. For instance, when a researcher decides to utilize the JSE (health professional version) [22,36], it is important to select interventions corresponding to the specific empathy dimensions (cognitive and affective dimensions) and the KAB aspects (the attitude aspect) in order to create consistent results. Thus, an effective intervention such as perspective taking, which targets the cognitive and affective dimensions, should be adopted.

Similarly, when an intervention is chosen, it is recommended that a measure with the corresponding empathy dimensions and KAB aspects be employed. For example, an intervention of a communication-associated workshop or educational course, which fulfills the behavioral dimension of empathy and the behavior aspect of the KAB model, should be assessed with a measure involving both the behavioral dimension and the behavior aspect, such as the ECCS [20] or Consultation and Relational Empathy (CARE) [31], instead of a measure having different dimensions and aspects, such as the JSE [22,36].

### 4.5. Limitations

The first limitation of this study concerns the categorization of each intervention of the selected studies. Ambiguity and difficulties in decisions were sometimes encountered even after thorough discussions among the authors. A strict policy was enacted so that only explicit statements were accounted for when determining the dimensions or aspects of the KAB model. Therefore, some dimensions or aspects may have been missed due to a lack of mention in specific studies, and such omissions may have affected our results.

The second limitation concerns the categorization of each measure that is applied in the selected studies. As with the first limitation, descriptions of some item questions were unclear, and only items with explicit statements were accounted for when determining the dimensions or KAB aspects. Hence, some dimensions or aspects may have been missed. Such omissions may have influenced the results of the categorization of certain measures and thus our findings.

The third limitation also concerns the categorization of measures. A measure that consists of more items is more likely to exceed the threshold number (three in our study) or proportion (one third of all items) that are required for categorization than a measure with fewer items. Therefore, for a measure with fewer items, certain dimensions or aspects may have been missed due to the inclusion of only a few (≤two) items.

## 5. Conclusions

General inconsistency was found among the sections of current medical empathy intervention studies with a randomized controlled trial design, either in terms of empathy dimensions or knowledge–attitude–behavior aspects. Behavioral components had the best consistency in each category. Researchers of a future empathy intervention study should confirm the consistency in terms of either empathy dimensions or knowledge–attitude–behavior aspects in order to achieve an appropriate outcome.

## Figures and Tables

**Table 1:** Compositions of empathy dimensions and knowledge–attitude–behavior (KAB) aspects among different sections of the medical empathy intervention studies (*N* = 13).

No.	Studies	Definition Section			Intervention Section						Assessment Section						Measures
		CE	AE	BE	CE	AE	BE	K	A	B	CE	AE	BE	K	A	B	
1.	Bonvicini et al., 2009 [18]	CE	AE	BE	--	--	BE	--	--	B	--	--	BE	--	--	B	GRS [19] ECCS [20]
2.	Bunn and Terpstra 2009 [21]	CE	AE	--	--	--	--	--	--	--	--	--	--	--	A	--	JSE-S [22] *
3.	Blatt et al., 2010 [23]	--	AE	--	CE	AE	--	--	--	--	--	--	BE	--	--	B	Clinical skills examinations [24,25,26]
4.	Tulsky et al., 2011 [27]	--	--	--	--	--	BE	--	--	B	CE	AE	BE	--	--	B	Emotion-handling skills [28,29,30] CARE [31]
5.	Riess et al., 2012 [32]	CE	AE	BE	--	AE	BE	K	--	B	CE	AE	BE	K	A	B	CARE [31] JSE-HP [22] * BEES [33] Neurobiology test [32]
6.	Potash et al., 2014 [34]	--	--	--	--	--	--	--	--	--	--	--	--	--	A	--	JSE-S [35,36] *
7.	Foster et al., 2016 [37]	CE	AE	BE	--	--	BE	--	--	B	--	--	BE	--	--	B	ECCS [20]
8.	Buffel du Vaure et al., 2017 [38]	--	AE	BE	--	--	--	--	--	--	CE	AE	BE	--	A	B	CARE [31] JSE-S (France version) [39] *
9.	LoSasso et al., 2017 [40]	--	--	--	--	--	BE	K	--	B	CE	AE	--	--	A	--	JSE-HP [22,36] * JSPPPE [41]
10.	Wündrich et al., 2017 [42]	CE	AE	BE	CE	--	BE	K	--	B	--	--	BE	--	A	B	SP-rated questionnaire [42] JSE-S (German version) [43] *
11.	Lemogne et al., 2020 [44]	CE	AE	BE	--	--	--	--	--	--	--	--	--	--	A	--	JSE-S (France version) [39] *
12.	Chen et al., 2021 [45]	CE	AE	--	--	--	--	--	--	--	CE	AE	BE	--	A	B	LCSAS [46,47] JSE-HP [22] *
13.	Grossman et al., 2021 [48]	--	--	--	--	--	BE	--	--	B	--	--	BE	--	--	B	ECCS [20]
Total		7	9	6	2	2	7	3	0	7	5	5	9	1	8	9	

**Abbreviations:** Legends: CE = cognitive empathy; AE = affective empathy; BE = behavioral empathy; K = knowledge; A = attitude; B = behavior. Measures: BEES = The Balanced Emotional Empathy Scale. CARE = Consultation and Relational Empathy. ECCS = Empathy Communication Coding System. GRS = Global rating scale. IRI = Interpersonal Reactivity Index. JSE-HP = Jefferson Scale of Empathy–health professional version. JSE-S = Jefferson Scale of Empathy–medical student version. JSPPPE = Jefferson scale of patient perceptions of physician empathy. LCSAS = Liverpool Communication Skills Assessment Scale. PCAS = Primary Care Assessment Survey. SP = simulated patient. --: Absent. *: Whereas the JSE-HP was categorized as having both “cognitive” and “affective” dimensions, the JSE-S was categorized as having “none” in terms of the empathy dimension due to a slight change in the wording of the questionnaire. For example, in many questions in the JSE-S, the subjects were changed from “I” to “physicians”, and hence the targets of measure were changed from “medical students themselves” to “physicians’ images” that were perceived by the students. Therefore, based on our definition, the dimension of the questions was then changed from either “cognitive” or “affective” to “none”, and thus the categorization of the measure.

**Table 2:** Percentage of consistency in terms of empathy dimensions and knowledge–attitude–behavior (KAB) aspects among different sections of the medical empathy intervention studies. (*N* = 13).

		Empathy Dimensions				KAB Aspects
No.	Studies	Overall	Definition vs. Intervention	Intervention vs. Assessment	Definition vs. Assessment	Intervention vs. Assessment
1.	Bonvicini et al., 2009 [18]	33%	33%	100%	33%	100%
2.	Bunn and Terpstra 2009 [21]	0%	0%	--	0%	0%
3.	Blatt et al., 2010 [23]	0%	50%	0%	0%	0%
4.	Tulsky et al., 2011 [27]	0%	0%	33%	0%	100%
5.	Riess et al., 2012 [32]	67%	67%	67%	100%	67%
6.	Potash et al., 2014 [34]	--	--	--	--	0%
7.	Foster et al., 2016 [37]	33%	33%	100%	33%	100%
8.	Buffel du Vaure et al., 2017 [38]	0%	0%	0%	67%	0%
9.	LoSasso et al., 2017 [40]	0%	0%	0%	0%	0%
10.	Wündrich et al., 2017 [42]	33%	67%	50%	33%	33%
11.	Lemogne et al., 2020 [44]	0%	0%	--	0%	0%
12.	Chen et al., 2021 [45]	0%	0%	0%	67%	0%
13.	Grossman et al., 2021 [48]	0%	0%	100%	0%	100%

## Data Availability

The datasets of our study are available on reasonable request.

## Appendix A. Search Terms and Strategies

PubMed:

“medical student*”[Title/Abstract] OR “physician*”[Title/Abstract] OR “surgeon*”[Title/Abstract] OR “doctor*”[Title/Abstract] OR “resident*”[Title/Abstract]) AND (“Empathy”[MeSH Terms] OR “empath*”[Title/Abstract]) AND (“Controlled Clinical Trial”[Publication Type] AND “random*”[Title/Abstract]

Scopus:

TITLE-ABS-KEY (“empath*”) AND TITLE-ABS-KEY (“intervention” OR “train”) AND TITLE-ABS-KEY (“doctor” OR “medical student” OR “physician” OR “surgeon*” OR “resident*”) AND TITLE-ABS-KEY (“randomized controlled trial”) AND (LIMIT-TO (PUBSTAGE, “final”)) AND (LIMIT-TO (DOCTYPE, “ar”))

## Appendix B. Categorization Criteria of Empathy Dimensions in the Definition, Intervention, and Assessment Sections

Definition section:

- Cognitive empathy was considered as knowing or understanding others’ thoughts or thought processes.
- Affective empathy was considered as understanding or recognizing others’ feelings or emotional states.
- Behavioral empathy was considered as performing actions, behaviors, or responses for showing understanding toward others.

Intervention section:

- Cognitive empathy was considered as teaching participants how to understand or recognize others’ thoughts, perspectives, or thought processes.
- Affective empathy was considered as teaching participants how to understand or recognize others’ feelings or emotional states.
- Behavioral empathy was considered as teaching participants how to communicate with others or how to show their understanding to others.

Assessment section:

- A measure was classified as assessing cognitive empathy if its item(s) aimed to evaluate or estimate one’s ability or tendency to understand or recognize others’ thoughts or thought processes.
- A measure was classified as assessing affective empathy if its item(s) aimed to evaluate or estimate one’s ability or tendency to understand or recognize others’ feelings or emotional states.
- A measure was classified as assessing behavioral empathy if its item(s) aimed to evaluate actions or behaviors that were \performed in order to show understanding to others.

## Appendix C. Categorization Criteria of Knowledge–Attitude–Behavior (KAB) Aspects in Intervention and Assessment Sections

Intervention section:

- The knowledge aspects include various forms of education delivering relevant knowledge of empathy, such as the definition of empathy, theory of empathy, or neural basis of empathy.
- The attitude aspects include activities promoting the importance of empathy or beliefs about empathy. Example activities include group discussions or lectures improving beliefs about and the importance of empathy.
- The behavior aspects include activities providing principles or practical methods to enhance empathic responses to others. Example activities include empathic communication skill workshops or emotion recognition training classes.

Assessment section:

- The knowledge aspects include item(s) evaluating one’s knowledge of empathy, such as the definition of empathy.
- The attitude aspects include item(s) evaluating one’s beliefs about or attitudes toward empathy.
- The behavior aspects include item(s) evaluating one’s expression of empathy.
